# Правовые аспекты обеспечения детей с сахарным диабетом 1 типа средствами непрерывного мониторинга глюкозы

**DOI:** 10.14341/probl13264

**Published:** 2023-08-30

**Authors:** О. А. Малиевский, С. А. Юлчурин

**Affiliations:** Башкирский государственный медицинский университет; Государственная клиническая больница № 5 г. Уфы

**Keywords:** сахарный диабет 1 типа, дети, непрерывный мониторинг глюкозы

## Abstract

Непрерывный мониторинг глюкозы является эффективным методом мониторинга гликемии, позволяющим достичь оптимального гликемического контроля и снизить риск осложнений сахарного диабета 1 типа у детей. После утверждения нового «Стандарта медицинской помощи детям при сахарном диабете 1 типа (диагностика и лечение)» (зарегистрирован в Минюсте России 18.02.2021 №62543) во многих регионах страны были инициированы сотни судебных исков к региональным органам государственной власти в области здравоохранения и медицинским организациям с требованием бесплатного предоставления систем непрерывного мониторирования уровня глюкозы в крови и/или датчиков к ним. В данной статье приведен анализ действующего законодательства в части обоснованности требований бесплатного обеспечения детей-инвалидов с сахарным диабетом 1 типа средствами непрерывного мониторинга глюкозы.

## Abstract (en)

Continuous glucose monitoring is an effective method of monitoring glycaemia, allowing to achieve optimal glycemic control and reduce the risk of complications of type 1 diabetes mellitus in children. After the approval of the new «Standard of medical care for children with type 1 diabetes (diagnosis and treatment)» (registered with the Ministry of Justice of Russia on 02/18/2021 No. 62543), hundreds of lawsuits were initiated in many regions of the country against regional public authorities in the field of healthcare and medical organizations demanding free supply of systems for continuous monitoring of blood glucose levels and/or sensors to them. This article analyzes the current legislation regarding the validity of the claims for free supply of disabled children with type 1 diabetes with continuous glucose monitoring.

С целью достижения оптимального гликемического контроля и снижения риска осложнений сахарного диабета 1 типа (СД1) необходим регулярный контроль глюкозы с использованием глюкометров или систем непрерывного мониторирования глюкозы (НМГ) [1]. Применение профессионального НМГ при оказании медицинской помощи детям с СД1 в амбулаторных условиях статистически значимо улучшает показатели гликемического контроля, определяемые по уровню гликированного гемоглобина (НbА1с), и среднего уровня гликемии. Снижение концентрации НbА1с сопровождается статистически значимым увеличением числа пациентов с оптимальным гликемическим контролем [2][3]. При переводе пациентов на помповую инсулинотерапию при регулярном использовании НМГ также отмечается лучший ответ [4].

Неизбежная сторона усилий по достижению целевых показателей самоконтроля, особенно НbА1с менее 7% — рост гипогликемических состояний. По данным Московского сегмента Федерального регистра сахарного диабета, отмечается тенденция к увеличению частоты тяжелых гипогликемий. Так, среди детей распространенность тяжелых гипогликемий за период с 2017 по 2020 гг. увеличилась в 12,3 раза (с 0,04 до 0,49%), а среди подростков — в 4,2 раза (с 0,2 до 0,8%). Рост числа пациентов с тяжелыми гипогликемиями ни в коей мере не исключает необходимости достижения целей гликемического контроля, но требует применения современных средств мониторинга гликемии [5].

В настоящее время количество пациентов, использующих НМГ, продолжает быстро увеличиваться, и эффективное внедрение этого метода в клиническую практику станет центральным элементом мониторинга за детьми и подростками с СД1 [6].

Применение НМГ предусмотрено клиническими рекомендациями «Сахарный диабет 1 типа у детей», в которых указано, что НМГ следует рассмотреть у пациентов при уровне HbA1c выше индивидуального целевого показателя; тяжелых гипогликемиях (≥1 раза за последний год); высокой вариабельности гликемии независимо от уровня НbА1с; частых эпизодах легкой гипогликемии; выраженном снижении качества жизни; при времени в целевом диапазоне менее 70% по данным НМГ [1].

Министерством здравоохранения Российской Федерации также был издан приказ №22н от 22.01.2021 г., которым утвержден новый стандарт медицинской помощи детям при СД1 (диагностика и лечение) (зарегистрирован в Минюсте России 18.02.2021 №62543) (далее — Приказ МЗ РФ №22н) [7]. Данным Приказом в раздел 2.2 «Лабораторные методы исследования» раздела 2 «Медицинские услуги для лечения заболевания, состояния и контроля за лечением» включено исследование уровня глюкозы в крови методом непрерывного мониторирования (A09.05.023.001).

После введения в действие с 01 марта 2021 г. вышеуказанного приказа во многих регионах страны были инициированы сотни судебных исков к региональным органам государственной власти в области здравоохранения и медицинским организациям с требованием бесплатного предоставления систем НМГ в крови и/или датчиков к ним.

Суды различных уровней удовлетворяют эти требования, определяя, что системы НМГ относятся к числу изделий медицинского назначения, которые должны быть предоставлены по жизненно необходимым показаниям ребенку-инвалиду с СД1 бесплатно за счет соответствующих региональных бюджетных средств или средств медицинского учреждения.

В связи с вышеизложенным мы сочли необходимым провести анализ действующего законодательства по поводу бесплатного обеспечения детей-инвалидов с СД1 средствами НМГ.

Доступность и качество медицинской помощи обеспечиваются в том числе применением порядков оказания медицинской помощи, клинических рекомендаций и стандартов медицинской помощи и предоставлением медицинской организацией гарантированного объема медицинской помощи в соответствии с программой государственных гарантий бесплатного оказания гражданам медицинской помощи (ст. 10 Федерального закона от 21.11.2011 №323-ФЗ (ред. от 26.03.2022) «Об основах охраны здоровья граждан в Российской Федерации») [8].

В ст. 80 (ч. 2) Федерального закона от 21.11.2011 №323-ФЗ «Об основах охраны здоровья граждан в Российской Федерации« говорится, что «…при оказании в рамках программы государственных гарантий бесплатного оказания гражданам медицинской помощи осуществляется обеспечение граждан медицинскими изделиями, включенными в утвержденный Правительством Российской Федерации Перечень медицинских изделий, имплантируемых в организм человека [8]. Порядок формирования данного перечня устанавливается Правительством Российской Федерации». Медицинские изделия, включенные в Перечень, не подлежат оплате за счет личных средств граждан при наличии клинических рекомендаций с учетом стандартов медицинской помощи. Перечень медицинских изделий, имплантируемых в организм человека при оказании медицинской помощи в рамках программы государственных гарантий бесплатного оказания гражданам медицинской помощи, утвержден Распоряжением Правительства РФ от 31.12.2018 №3053-р (ред. от 14.01.2022) [9]. Средства НМГ в данном перечне отсутствуют.

Также не подлежит оплате за счет личных средств граждан применение по медицинским показаниям медицинских изделий, не входящих в перечень медицинских изделий, имплантируемых в организм человека, но назначаемых по жизненным показаниям решением врачебной комиссии (ч. 3 ст. 80 №323-ФЗ) [8]. В связи с этим необходимо отметить, что в имеющейся в открытом доступе Инструкции производителя по применению «Датчика FreeStyle Libre системы Flash-мониторинга глюкозы FreeStyle Libre» указаны ситуации, в которых нельзя полагаться на показания данной системы: во время быстрых изменений уровня глюкозы, когда показания датчика FreeStyle Libre, основанные на содержании глюкозы в интерстициальной жидкости, могут не точно отражать текущий уровень глюкозы в крови; когда уровень глюкозы быстро снижается, показания датчика FreeStyle Libre могут оказаться выше, чем текущий уровень глюкозы в крови. И наоборот, когда уровень глюкозы быстро повышается, показания датчика FreeStyle Libre могут оказаться ниже, чем текущий уровень глюкозы в крови; когда показания датчика FreeStyle Libre указывают на гипогликемию или угрозу ее развития.

Значительное обезвоживание и существенная потеря воды, которые, как правило, развиваются при декомпенсации сахарного диабета, также могут привести к недостоверным результатам.

Таким образом, при неотложных, угрожающих жизни состояниях, таких как гипергликемия и гипогликемия, при проведении инсулинотерапии необходимо ориентироваться только на показания глюкометра, а не системы мониторинга. Следовательно, сам производитель ориентирует потребителя, что их датчики не назначаются по жизненным показаниям.

Еще одним законом, определяющим права на бесплатное обеспечение медицинскими изделиями, является Федеральный закон №178-ФЗ «О государственной социальной помощи» от 17.07.1999 г. [10]. Статьей 6.1 (п. 9) данного закона установлено, что дети-инвалиды имеют право на получение государственной социальной помощи в виде набора социальных услуг, который включает обеспечение необходимыми медицинскими изделиями по рецептам за счет средств федерального бюджета. Для бесплатного оказания гражданам медицинской помощи в рамках программы государственных гарантий статьей 6.2 (ч. 2) Закона №178-ФЗ определено, что Правительство Российской Федерации утверждает перечень медицинских изделий, перечень специализированных продуктов лечебного питания для детей-инвалидов, обеспечение которыми осуществляется в соответствии с пунктом 1 части 1 настоящей статьи, и порядки формирования таких перечней. Распоряжением Правительства РФ от 31.12.2018 г. №3053-р (ред. от 14.01.2022 г.) утвержден Перечень медицинских изделий, имплантируемых в организм человека, отпускаемых по рецептам на медицинские изделия при предоставлении набора социальных услуг (далее Перечень) [9]. В данном перечне средства НМГ отсутствуют.

В большинстве судебных исков пациенты апеллируют к Постановлению Правительства РФ от 30.07.1994 №890 «О государственной поддержке развития медицинской промышленности и улучшении обеспечения населения и учреждений здравоохранения лекарственными средствами и изделиями медицинского назначения» [11]. В «Перечень групп населения и категорий заболеваний, при амбулаторном лечении которых лекарственные средства и изделия медицинского назначения отпускаются по рецептам врачей бесплатно», утвержденный данным Постановлением, включены дети-инвалиды. Им бесплатно предоставляются все лекарственные средства, средства медицинской реабилитации, калоприемники, мочеприемники и перевязочные материалы. В перечень категорий заболеваний включен диабет, при наличии которого предоставляются все лекарственные средства, этиловый спирт (100 г в месяц), инсулиновые шприцы, шприцы типа «Новопен», «Пливапен» 1 и 2, иглы к ним, средства диагностики. Средства для НМГ в Перечне отсутствуют. Однако пациенты требуют бесплатного обеспечения средствами НМГ, мотивируя тем, что они являются средствами диагностики.

Однако в разделе 1 «Медицинские услуги для диагностики заболевания, состояния» «Стандарта медицинской помощи детям при сахарном диабете 1 типа (диагностика и лечение)», утвержденного приказом Министерства здравоохранения Российской Федерации от 22 января 2021 г. N22н, средства НМГ отсутствуют [7]. Таким образом, они не могут быть отнесены к средствам диагностики.

Более того, в Инструкции по применению медицинского изделия «Датчик FreeStyle Libre системы Flash-мониторинга глюкозы FreeStyle Libre» указано, что ухаживающее лицо должно либо самостоятельно проводить у ребенка измерения с помощью системы FreeStyle Libre и интерпретировать полученные результаты, либо оказывать ребенку помощь в проведении измерений и интерпретации результатов. В клинических рекомендациях «Сахарный диабет 1 типа у детей» НМГ нецелесообразен при отсутствии возможности и способности пациента и/или законного представителя активно использовать НМГ [1]. Таким образом, НМГ не является медицинской услугой, так как в соответствии с инструкцией он проводится не медицинским работником или иным работником, имеющими право на осуществление медицинской деятельности, а ухаживающим лицом или самим пациентом. Врач детский эндокринолог лишь обсуждает результаты мониторинга c пациентом/ухаживающим лицом и дает рекомендации по ведению пациента. Это осуществляется в рамках медицинских услуг B01.058.003 — прием (осмотр, консультация) врача детского эндокринолога первичный и B01.058.004 — прием (осмотр, консультация) врача детского эндокринолога повторный. Таким образом, НМГ не является медицинской услугой как таковой. Более того, услуга A09.05.023.001 именуется как исследование уровня глюкозы в крови методом непрерывного мониторирования, тогда как, согласно Инструкции по применению медицинского изделия «Датчик FreeStyle Libre системы Flash-мониторинга глюкозы FreeStyle Libre», данный датчик предназначен для измерения уровня глюкозы в интерстициальной жидкости, а не в крови.

Таким образом, на наш взгляд, правовые основания для производства госзакупок отсутствуют. Вместе с тем вступившие в законную силу решения судов накладывают на региональные органы государственной власти в области здравоохранения, а также медицинские учреждения первого звена исполнение обязанностей, которые в соответствии с федеральным законодательством относятся к ведению Российской Федерации.

В связи с этим считаем необходимым обратить внимание на то, что финансирование оказания государственной социальной помощи в виде набора социальных услуг предусматривается в виде субвенций из федерального бюджета (ч. 2 статьи 4.1. Закона №178-ФЗ) [10]. Средства на осуществление полномочий носят целевой характер и не могут быть использованы на другие цели, а в случае использования средств не по целевому назначению федеральный орган исполнительной власти, осуществляющий функции по контролю и надзору в финансово-бюджетной сфере, вправе взыскать указанные средства в порядке, установленном законодательством Российской Федерации (чч. 4–6 ст. 4.1).

Финансовое обеспечение расходных обязательств Российской Федерации по предоставлению гражданам государственной социальной помощи осуществляется в виде набора социальных услуг (далее — социальные услуги), включающего, в частности, обеспечение пациентов в соответствии со стандартами медицинской помощи необходимыми лекарственными препаратами для медицинского применения, медицинскими изделиями, а также специализированными продуктами лечебного питания для детей-инвалидов. Порядок этого обеспечения определяется Постановлением Правительства РФ от 29.12.2004 №864 «О порядке финансового обеспечения расходов по предоставлению гражданам государственной социальной помощи в виде набора социальных услуг» [12].

Пунктом 4 вышеупомянутого Постановления определено, что финансовое обеспечение расходов, связанных с предоставлением социальных услуг, осуществляется за счет средств, предусмотренных федеральным законом о федеральном бюджете на соответствующий финансовый год и плановый период для Министерства здравоохранения Российской Федерации — на реализацию мер социальной поддержки отдельных категорий граждан по обеспечению в соответствии со стандартами медицинской помощи необходимыми лекарственными препаратами для медицинского применения по рецептам на лекарственные препараты, медицинскими изделиями по рецептам на медицинские изделия, а также специализированными продуктами лечебного питания для детей-инвалидов (далее — обеспечение граждан лекарственными препаратами).

Следует дополнительно отметить, что в соответствии с Федеральным законом №178-ФЗ «О государственной социальной помощи» оказание государственной социальной помощи предполагает не только предоставление набора социальных услуг, но и денежные выплаты в виде социального пособия, которые регулируются Федеральным законом от 24.11.1995 г. №181-ФЗ (ред. от 28.06.2021 г.) «О социальной защите инвалидов в Российской Федерации», в котором в статье 2 указано, что социальная защита инвалидов — это система гарантированных государством экономических, правовых мер и мер социальной поддержки, обеспечивающих инвалидам условия для преодоления, замещения (компенсации) ограничений жизнедеятельности и направленных на создание им равных с другими гражданами возможностей участия в жизни общества [13]. Именно этим целям и соответствует использование НМГ у детей с СД1.

В своих судебных исках и обращениях в прокуратуру все без исключения пациенты умалчивают факт получения данного пособия, заявляют, что приобретают средства НМГ за счет собственных средств, и на этом основании требуют компенсации за затраченные средства. Однако сумма выдаваемого инвалидам пособия существенно превышает расходы на приобретение средств НМГ.

Таким образом, в настоящее время отсутствует законодательная база, предусматривающая обязательства бюджетов различного уровня по бесплатному обеспечению детей с СД1 средствами НМГ.

Ст. 5 (п. 6) Федерального закона от 24.11.1995 №181-ФЗ (ред. от 28.06.2021) «О социальной защите инвалидов в Российской Федерации» гласит, что органы государственной власти субъектов Российской Федерации в области социальной защиты и социальной поддержки инвалидов имеют право предоставления дополнительных мер социальной поддержки инвалидам за счет средств бюджетов субъектов Российской Федерации. В некоторых субъектах используются различные механизмы обеспечения детей с СД1 средствами НМГ за счет региональных бюджетов. В частности, в Республике Башкортостан Постановлением Правительства республики от 6 сентября 2022 г. №513 «Об установлении дополнительных мер социальной поддержки по обеспечению медицинскими изделиями детей, страдающих сахарным диабетом 1 типа» предусмотрено выделение средств из республиканского бюджета. Во многих субъектах такая возможность отсутствует, что создает социальное неравенство в отношении детей-инвалидов в зависимости от места проживания.

## ЗАКЛЮЧЕНИЕ

Наиболее рациональным, на наш взгляд, является включение средств НМГ в «Перечень медицинских изделий, имплантируемых в организм человека, отпускаемых по рецептам на медицинские изделия при предоставлении набора социальных услуг».

## ДОПОЛНИТЕЛЬНАЯ ИНФОРМАЦИЯ

Информация о конфликте интересов. Авторы декларируют отсутствие явных и потенциальных конфликтов интересов, связанных с публикацией настоящей статьи.

Информация о финансировании. Работа выполнена по инициативе авторов без привлечения финансирования.

Информация о вкладе каждого автора. Малиевский О.А. — поиск литературных источников, написание текста рукописи; Юлчурин  С.А. — анализ законодательных и нормативных актов. Все авторы одобрили финальную версию статьи перед публикацией, выразили согласие нести ответственность за все аспекты работы, подразумевающую надлежащее изучение и решение вопросов, связанных с точностью или добросовестностью любой части работы.
